# Simplification of Rabies Postexposure Prophylaxis: A New 2-Visit Intradermal Vaccine Regimen

**DOI:** 10.4269/ajtmh.19-0252

**Published:** 2019-08-05

**Authors:** Mary J. Warrell

**Affiliations:** Oxford Vaccine Group, University of Oxford, Oxford, United Kingdom

## Abstract

A 2-visit multiple-site intradermal (ID) vaccine protocol would be the most economical, immunogenic, and practicable regimen for postexposure rabies prophylaxis (PEP) in clinics seeing few patients a month. This regimen with an additional day 28 dose is now recommended by the WHO. The difficulties surrounding ID rabies vaccination have hindered progress in provision of prophylaxis, especially in rural Asia and Africa. Although the latest WHO recommendations include 1-week ID postexposure vaccine regimens, these are unlikely to prove economical where rabies vaccination is presently unavailable. The new protocol uses a whole vial of vaccine divided between 4-sites ID on the first day and half a vial at 2-sites ID on day 7. Gavi has recently approved support for rabies PEP. This 2-visit 4-site ID regimen, with or without a day 28 dose, should be considered for implementation in this remarkable new initiative.

## THE CURRENT SITUATION

Rabies encephalitis following a rabid dog bite is always fatal in unvaccinated patients, yet correct preventive vaccination has proved 100% effective. Rabies vaccine is frequently unavailable or unaffordable in rural areas of Asia and Africa, where up to 90% of rabies deaths occur.^1^ Gavi, the Vaccine Alliance, recently approved support for human rabies vaccine for postexposure prophylaxis (PEP), beginning in 2021. The success of this endeavor will depend on whether the expensive vaccines can be provided economically. Using small doses of vaccine intradermally (ID) is highly immunogenic and economical.^2^

The WHO has recently recommended the new IPC (Institut Pasteur du Cambodge) postexposure vaccine regimen consisting of 0.1 mL ID injection at 2 sites on days 0, 3, and 7.^3^ This is the same as the method recommended for 20 years, the Thai Red Cross (TRC) 2-site ID regimen, but without the day 28 dose (Table 1). The WHO decision to accept this regimen was based on preliminary serological data from some of the patients in a clinical trial.^4^ (The study used vaccine containing 0.5 mL/vial.) The full data remain unavailable a year later.^2^ Rabies vaccines do not contain preservatives and their use ID is off-label but is sanctioned by the WHO, provided that an opened vial is used within 8 hours. Attempts to use the TRC regimen in rural clinics where only a few dog bite patients are treated each month have failed, mainly because of vaccine wastage. This regimen has proved economical only in urban clinics seeing several patients a week. It seems unlikely that the new 1-week IPC method will solve that problem. Rabies immunoglobulin, officially recommended for all but the most trivial bites,^3^ is not expected to be available.

**Table 1:** Selected WHO recommended postexposure rabies vaccine regimens*

Number of ID sites injected						
	Day 0	Day 3	Day 7	Day 28	Visits	Total vaccine
WHO principle ID recommendation						
ID 2-site 1-week “IPC”	2	2	2	–	3	Depends on vial size: 1–2 vials, 3 vials if no sharing
WHO alternative regimens						
ID 2-site Thai Red Cross	2	2	2	2	4	1–2 vials, 4 vials if no sharing
ID 4-site 1 month	4		2	1	3	< 2 vials, same for all vaccines, 3 vials if no sharing

ID = intradermal; IPC = Institut Pasteur du Cambodge.

* Six regimens were recommended: two intramuscular and four ID.

## TWO-VISIT ID 4-SITE POSTEXPOSURE VACCINE REGIMEN

Another regimen, the 2-visit ID 4-site, was not considered by the Strategic Advisory Group of Experts on Immunization or WHO, although the same regimen with an additional 3rd dose on day 28 has been recommended by the WHO as an option for PEP (see Table 1). It is derived from the original 8-site ID regimen,^5^ which was recommended by the WHO for several years, using 1 mL/vial vaccines.^6^ The 8-site regimen was compared with the 2-site ID TRC regimen using the same amount of vaccine antigen. The neutralizing antibody response induced by the 8-site was significantly higher from day 7 up to a year later.^7^ Dividing the large ID dose on day 0 between days 0 and 3, as in the 2-site TRC regimen, was thus shown to be less immunogenic. Furthermore, in 200 Indian patients given a whole vial of vaccine at eight ID sites on 1 day, there was universal seroconversion by day 14.^8^ The superior immunogenicity of the 8-site regimen was acknowledged by the WHO.^9^

The protocol of the 8-site ID regimen using 1 mL/vial vaccine was changed when rabies vaccines were produced with 0.5 mL/vial. The number of ID sites was halved and the dose per site doubled. This became the 4-site ID 1-month regimen which is now recommended by the WHO.^3^ A whole vial is divided between 4-sites ID on day 0, half a vial between 2-sites ID on day 7, and at one site on day 28 (Table 1).^10^

The ID dose for this regimen is 0.1 mL/site for 0.5 mL/vial vaccines and 0.2 mL/site for 1 mL/vial. If injecting 0.2 mL ID is difficult, the needle can be withdrawn and the remaining dose is given at an adjacent site. In this regimen, the amount of vaccine antigen remains constant, unlike in the 2-site regimens, which use an ID dose of 0.1 mL with any vaccine, thereby halving the dose with 1 mL/vial vaccines.^2^ Because the dosage and timing of these 8-site and 4-site ID methods are identical, the proven efficacy of the 8-site regimen in postexposure trials and in the field over many years also applies to the 4-site version.^5,11^

It is well established that the protective effect of PEP is due to the induction of neutralizing antibody within the first few days. Early immunogenicity following rabies vaccination is related to the initial amount of antigen injected ID.^11^ The day 28 dose is no longer deemed necessary as a routine because it is the early antibody that is most important in preventing fatal rabies encephalomyelitis. The resulting 2-visit 4-site ID regimen (Table 2) would be the most economical and suitable for rural Asian and African clinics. There is no vial sharing on day 0 as the first dose uses a whole vial. If some vaccine were accidentally injected subcutaneously, immunogenicity would not be jeopardized as half the dose has been shown to be immunogenic under trial conditions.^12^ Experience with ID injection technique is therefore not essential as there is a wide margin of safety, compared with the relative vulnerability of the 2-site ID IPC or TRC regimens.

**Table 2:** Simplified scheme for economical 4-site ID rabies postexposure vaccination

Number of ID sites injected						
	Day 0	Day 3	Day 7	Day 28	Visits	Total vaccine
Primary Postexposure Regimens						
2-visit 4-site PEP*	4†	–	2	–	2	1.5 vials, same for all vaccines, 2 vials if no sharing
2-visit 4-site PEP with 3rd dose for immunosuppressed patients, optional if healthy*	4†	–	2	1	3	< 2 vials, same for all vaccines, 3 vials if no sharing
Postexposure booster regimen for those previously immunized						
Single day, 4-site	4‡	–	–	–	1	0.5–1 vial

ID = intradermal; PEP = postexposure rabies prophylaxis.

* ID doses are 0.1 mL/site for 0.5 mL/vial vaccine (Purified Vero cell Rabies Vaccine, Verorab; Sanofi, Lyon, France) or 0.2 mL/site for 1.0 mL/vial vaccine (Purified Chick Embryo Cell Vaccine, Rabipur/RabAvert; GSK, Marburg, Germany).

† Use whole vial.

‡ Preferably using a whole vial especially if previously vaccinated several years before, but half vial can be used with 1-mL vaccines.

If sharing of vials is not possible with the 2-visit 4-site ID scheme, a total of two vials of vaccine are required, less than with any other regimen. An ID dose on day 28 is recommended for immunosuppressed patients after any 1-week PEP regimen.^2,3^

Although there has been no clinical trial of this 2-visit 1-week regimen, there are compelling comparative serological data for the same doses on days 0 and 7 with an additional day 28 single ID dose. The immunological data presented above on the 8-site /4-site ID 1-month regimen demonstrate 100% seroconversion by day 14.^5,7,8,10^ This is the same as with the 2-visit schedule (before the day 28 dose), and so it meets the WHO criterion for the efficacy of a PEP vaccine regimen. A phase IV observational study of the new regimen would give additional information on the economy and practicability and also, if serology were available, confirm the immunogenicity of the 2-visit PEP regimen. Among the WHO recommended regimens, the 4-site ID 1-month regimen can be implemented globally immediately. It is especially suitable if the patient is likely to default, there is uncertainty about the accuracy of ID injection, if vaccine is scarce or if the cost is prohibitive.

## SIMPLIFIED SCHEME FOR 4-SITE ID POSTEXPOSURE VACCINATION

A simple plan is proposed for all PEP using the same first dose (Table 2). For previously vaccinated patients, the WHO has long recommended a 4-site ID single-day postexposure booster regimen. Therefore, a 4-site ID dose on day 0 could be given to all patients exposed to a possibly rabid mammal. If they have never had vaccine before, a second 2-site ID dose is given on day 7. Immunosuppressed patients would be given an additional single-site ID dose on or around day 28.^2^

## CONCLUSION

The choice of postexposure regimen will influence whether widespread donation of expensive vaccines by Gavi is economically viable and can save thousands of lives.
